# Supplementary figures and images for: The Molecular Mechanism of a Cis-Regulatory Adaptation in Yeast
Source: PLoS Genet. 2013 Sep 19;9(9):e1003813. doi: 10.1371/journal.pgen.1003813 (PMC3778017; doi:10.1371/journal.pgen.1003813)

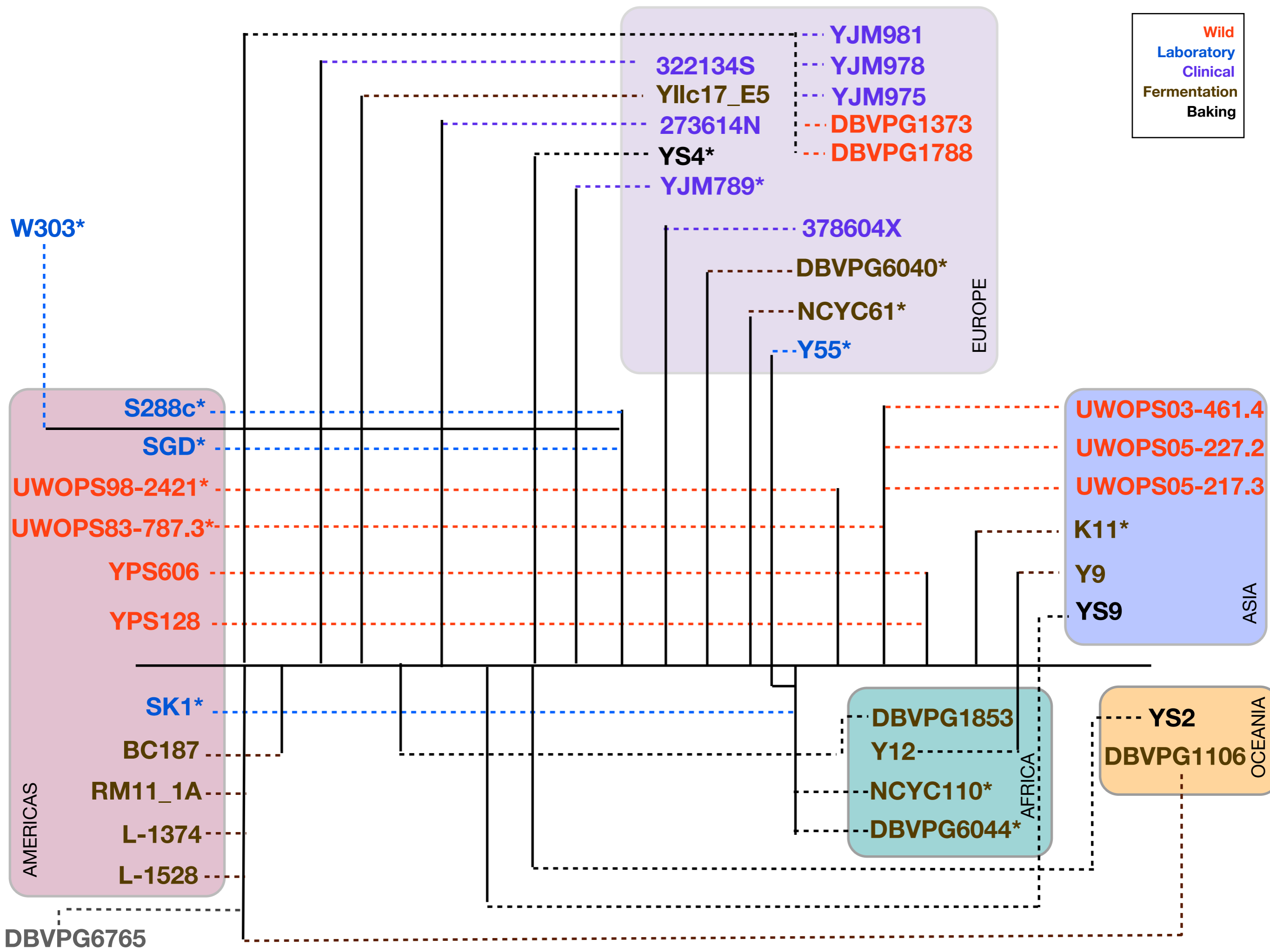

Supplement: Figure S1 — Phylogeny of S. cerevisiae (adapted from [37]), with strains containing the AA112Δ allele marked with an asterisk. Strains are grouped by geographic origin; branch lengths are not to scale. “SGD” and “S288c” are nearly identical to BY, so are not counted among the 12 non-BY strains with the AA112Δ variant. (PDF) [file pgen.1003813.s001.pdf]
